# Application of Y–Z deformable magnetic ring for recanalization of transanal single-access rectal stricture

**DOI:** 10.1038/s41598-024-52531-4

**Published:** 2024-01-22

**Authors:** Miaomiao Zhang, Yingying Zhuang, Jianqi Mao, Mingyan Gong, Yuhan Zhang, Aihua Shi, Yi Lyu, Xiaopeng Yan

**Affiliations:** 1 Department of Hepatobiliary Surgery, The First Affiliated Hospital of Xi’an Jiaotong University, No. 277 West Yanta Road, Xi’an, 710061 Shaanxi China; 2 National and Local Joint Engineering Research Center of Precision Surgery & Regenerative Medicine, The First Affiliated Hospital of Xi’an Jiaotong University, No. 76 West Yanta Road, Xi’an, 710061 Shaanxi China; 3 Baoji Central Hospital, Baoji, Shaanxi China; 4 Zonglian College, Xi’an Jiaotong University, Xi’an, 710061 Shaanxi China; 5 Qide College, Xi’an Jiaotong University, Xi’an, 710061 Shaanxi China

**Keywords:** Gastrointestinal system, Gastrointestinal diseases, Gastroenterology, Surgery

## Abstract

Magnetic compression anastomosis has been reported to have remarkable clinical outcomes. Here, we tested the applicability of a Y–Z deformable magnetic ring (DMR) for non-surgical manipulation of rectal stenosis (RS) in a beagle dog model under a transanal single-access condition. RS was modeled in 8 beagle dogs using partial ligation with silk thread. Under X-ray guidance, the Y–Z DMR was positioned at the proximal and distal ends of the RS, and the magnetic ring was bent into an “O” shape, such that the two rings were magnetically attracted. Operation time, complications during or after operation, and discharge time of the magnetic rings were recorded. The anastomosis bursting pressure was measured two weeks after removing the rings, and its formation was assessed through gross and histological examination. Partial ligation with a silk thread successfully established the canine RS model. After Y–Z DMR installation, the magnetic ring was successfully reconfigured from an “S” to an “O” shape. Strong attraction existed between the rings. The operation time was 9–15 min (average: 11.75 ± 1.98 min). No rectal bleeding or perforation occurred during or after operation. The ring was naturally expelled 7–10 days after surgery. A pressure of > 300 mmHg was recorded at the point of anastomosis rupture. The rectal anastomosis appeared to have healed properly on the surface, which was confirmed histologically, signifying the success of this procedure. A Y–Z DMR facilitated the successful recanalization of transanal single-channel RS without needing surgery in an animal model.

## Introduction

With an estimated 1.8 million new cases and about 881,000 deaths reported in the year 2018, colorectal cancer (CRC) is the third-most common cancer and the second leading cause of cancer-related deaths worldwide^1^. CRC treatment entails extensive measures, with surgery being the primary one^2,3^. The rate of sphincter preservation in patients with a low incidence of rectal cancer has been increasing alongside the development of resection techniques for low and ultra-low rectal cancer. Sphincter preservation rates have improved, but the occurrence of anastomosis stenosis (AS) is also prevalent. Approximately 6–10% of all patients are diagnosed with AS following rectal cancer surgery^4^. Some studies have reported that the incidence of AS ranges from 3 to 30%, with only 5% presenting with clinical symptoms. The discrepancy in prevalence estimates is likely attributed to the different diagnostic criteria used for AS^5–7^. Presently, mechanical dilatation, endoscopic balloon dilatation, endoscopic intestinal stent implantation, and endoscopic radial incision of anastomosis are all used in the clinical treatment of AS following rectal cancer surgery to rule out tumor-recurrence factors. Each of these treatments has its advantages and disadvantages. Patients with mild AS have a much better chance of achieving good therapeutic effects, while those with severe AS often face the enormous trauma of surgery and limited clinical benefits. Some patients with severe AS require a proximal colostomy or ileostomy because mechanical dilatation and endoscopic treatment have limited efficacy. Thus, research into less invasive yet highly effective treatment methods for patients with severe AS is of paramount importance.

The term “magnamosis” was coined in 2009 by Harrison^8^. After manual suture anastomosis (the first-generation anastomosis mode) and staple anastomosis (the second-generation anastomosis mode), magnamosis is the third-generation anastomosis mode, which is also known as smart anastomosis^9^. Pathological changes of adhesion-repair-healing were shown to occur in the tissues adjacent to the compression under the influence of “non-contact” magnetic field force, while ischemia, necrosis, and exfoliation occurred in the compressed tissues at the anastomosis site^10^. Magnamosis, which has been studied and applied in medicine for over 40 years, is finding increasing use in both academic and medical settings. Past researchers have found that magnamosis can be applied for any anastomosis involving a lumen in the digestive tract, including esophageal^11^, esophagogastric^12^, gastrojejunal^13,14^, small intestine^15^, colon^16^, and biliary-enteric anastomosis^17^. Magnamosis can be used for vascular anastomosis^18,19^ as well as for creating therapeutic^20^ and pathological^21^ fistulas.

In this study, animal experiments were performed to test the feasibility and anastomosis effect of the Y–Z deformable magnetic ring (Y–Z DMR) in the treatment of rectal stenosis under a single transanal pathway. We also considered the characteristics of AS after rectal cancer surgery and combined them with the principle of magnetic anastomosis.

## Materials and methods

### Ethics statement

This study was reviewed and approved by the Committee for Ethics of Animal Experiments of Xi’an Jiaotong University (approval number: 2022-1451). All experiments were performed in accordance with relevant guidelines and regulations for the Care and Use of Experimental Animals issued by the Xi’an Jiaotong University Medical Center. Eight beagle dogs (4 male, 4 female) aged > 1 year old and with bodyweights of 12–15 kg were provided by the Laboratory Animal Center of the Xi’an Jiaotong University (Xi’an China). The animals were acclimatized to laboratory conditions (23 °C, 12 h/12 h light/dark, 50% humidity, ad libitum access to food and water) for 1 week prior to commencing the experiments.

### Study design

This study is a feasibility verification experiment; hence, there was no control group, and all experimental animals were included in the study group. All dogs were adapted to single-cage feeding for 1 week after purchase. Under an open abdomen, the model of rectal stenosis was prepared through partial ligation with silk thread. The inner diameter of the rectum at the stenosis was approximately 4 mm. The Y–Z DMR was placed into the proximal and distal ends of the stenosis through the anus, and the state of the magnetic ring was observed and monitored using X-ray. X-ray examination was conducted every other day. Because beagle dogs were docile, so the X-ray examination was conducted under non-anesthesia. The operation time and magnetic ring discharge time were recorded. The surgical time defined here refers to the time spent on magnet placement, deformation, and anastomosis, excluding the time spent on model preparation and abdominal closure. The gross specimen of anastomosis was obtained to measure the bursting pressure of anastomosis, and healing of the anastomosis was observed by the naked eye and through light microscopy.

### Y–Z DMR

**Figure 1:**
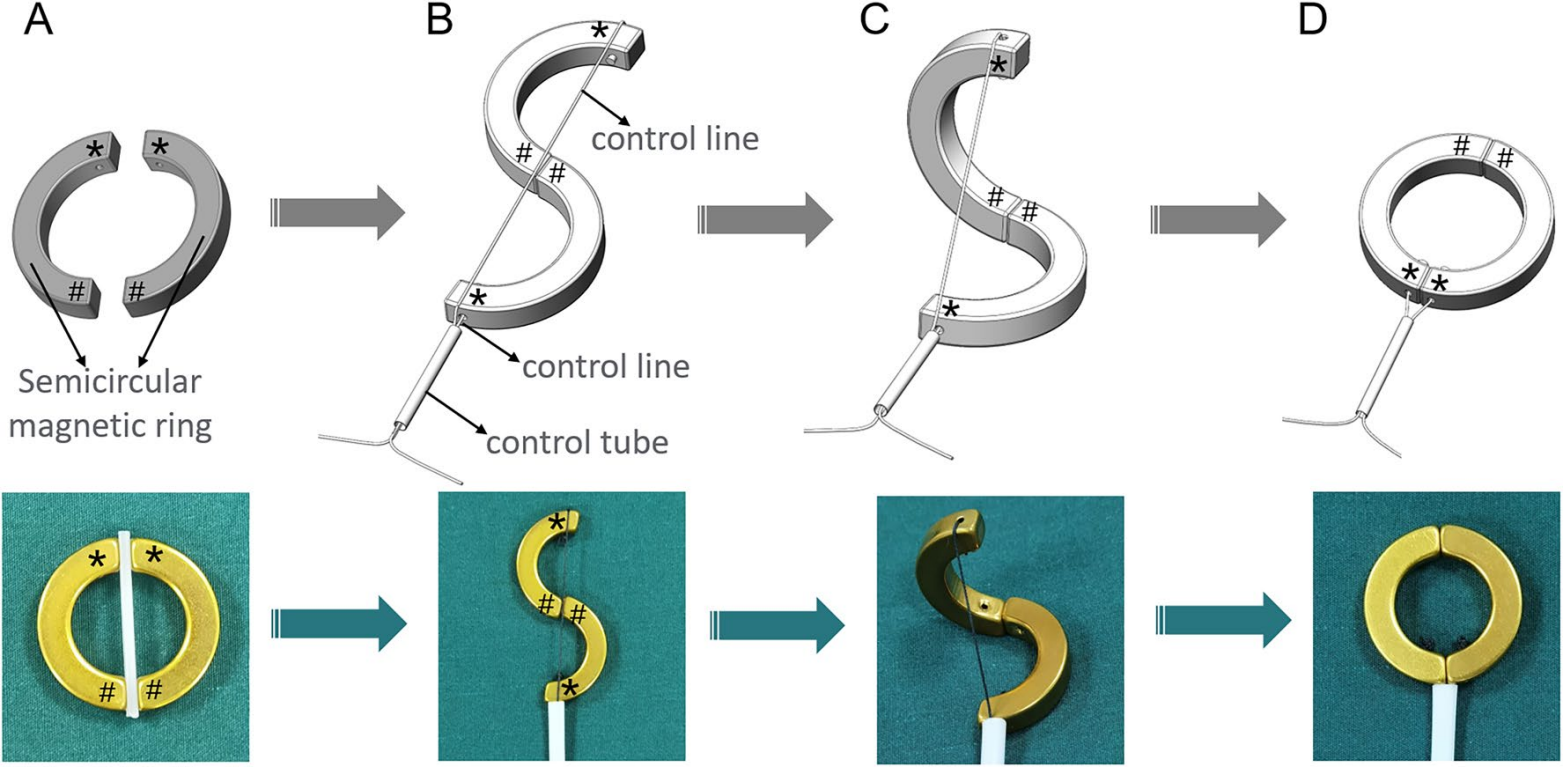
Schematic diagram depicting the Y–Z DMR process. (**A**) The semicircular magnetic ring. (**B**) Y–Z DMR in the “S”-shaped state. (**C**) The transition process of the Y–Z DMR from an “S” to “O” shape. (**D**) Y–Z DMR in the “O”-shaped state. *Control end, ^#^joint end.

**Figure 2:**
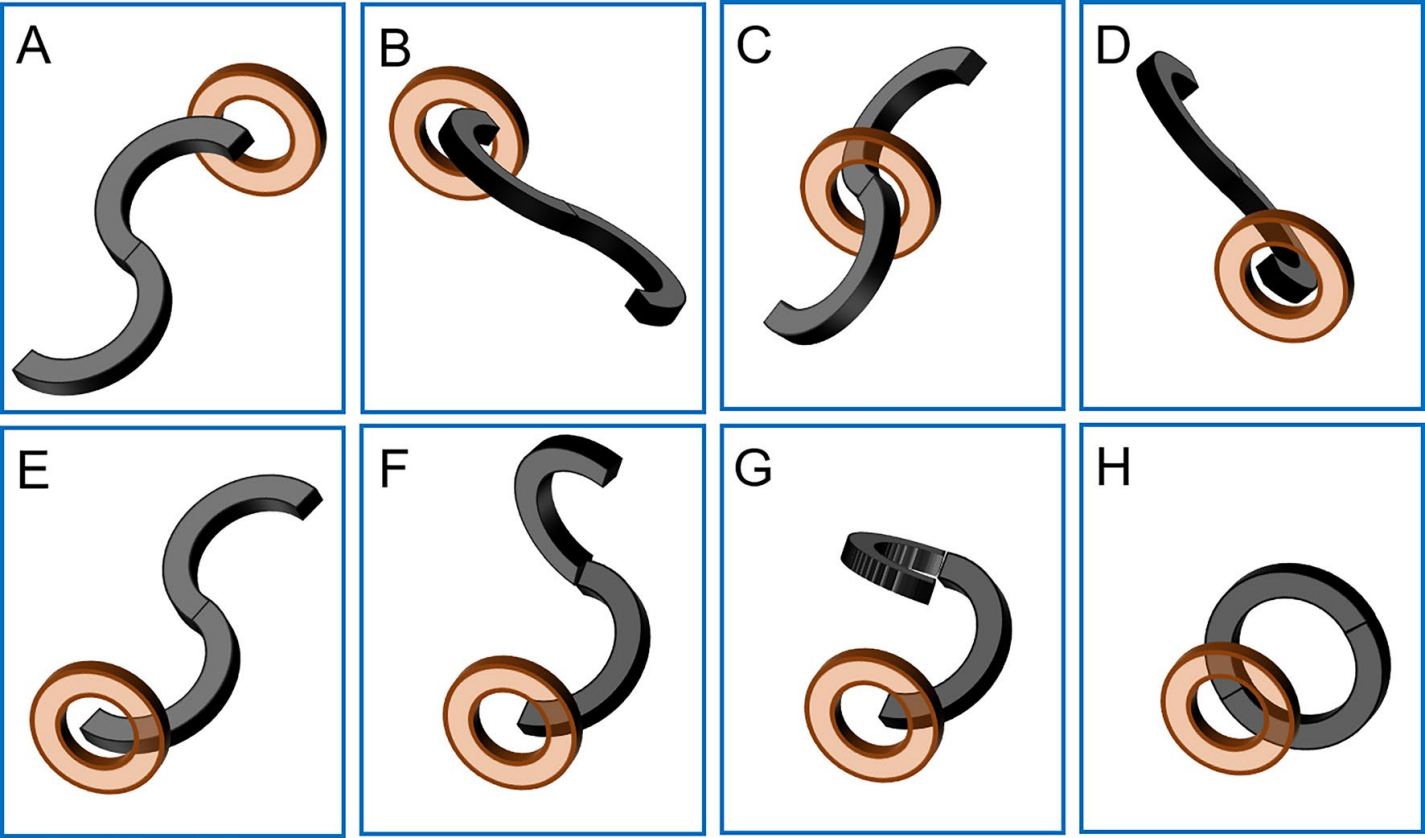
Schematic representation of the state of the Y–Z DMR through the narrow lumen. (**A**) The “S”-shaped state of the Y–Z DMR is close to the narrow segment. (**B**) The magnet is swung such that its head enters the narrow segment. (**C**) A semicircular magnetic ring passed through the narrow segment. (**D**) The magnet is swung, and the second semicircle is about to pass through the narrow section. (**E**) The entire “S”-shaped magnetic ring passes through the narrow segment. (**F**) The “S”-shaped state magnet begins to deform. (**G**) Magnets in the process of deformation. (**H**) The “S”-shaped magnet is completely transformed into an “O”-shaped one.

The Y–Z DMR consists of two semicircular magnetic rings (outer diameter: 32 mm, inner diameter: 22 mm, height: 5 mm). It is processed by N38-sintered NdFeB. The mass of each half ring was 7.83 g. The two ends of the semicircle magnetic ring are called the control end and the joint end, respectively, and the control end was used to connect the control line (1-0 silk wire) by adhesive (Fig. 1A). The joint ends of the two semicircle magnetic rings were attracted together, and the two control ends were respectively fixed on both sides of the joint end after the control line was fixed, while the two control lines passed through the control tube (Fig. 1B). The control tube was fixed, the control line of the distal semicircle magnetic ring was pulled, and the distal semicircle magnetic ring could rotate around the joint end until the control ends of the two semicircle magnetic rings were attracted to each other. At this point, the Y–Z DMR completed the transformation from “S” to “O” (Fig. 1C,D). Figure 2A–H illustrates the process of the Y–Z DMR traversing the stenosis and transforming from the “S” shape to the “O” shape in a serpentine motion. The Y–Z DMR was designed by Yan Xiaopeng (the corresponding author) and Zhang Miaomiao (the first author), and manufactured by Jinshan Electronic Appliances LTD. (Xi’an, China).

### Canine model of rectal stenosis

The dogs were anesthetized by intravenous administration of 30 mg/kg pentobarbital sodium solution and then placed in a supine position on an operating table with their abdomen shaved and sterilized. We carried out the operation with tracheal intubation and anesthesia machine after general anesthesia. The intraoperative oxygen concentration was 100%, respiratory rate was 12 times/min, and tidal volume was 12 ml/kg. The vital signs were observed by ECG monitor during operation. An intravenous channel was established, 500 ml sodium lactate Ringer’s solution was slowly administered intravenously and 0.5 g prophylactic antibiotic cefotiam was used. An electric insulation blanket was used to maintain appropriate body temperature. The abdominal cavity was accessed through an 8-cm long midline lower abdominal incision in the skin.

After the abdominal cavity was found to be normal, the rectum was searched distal along the descending colon, and a 14-Fr silicone tube was inserted into the colon through the anus. At the beginning of the rectum, the mesorectum was separated and the rectum was partially ligated into a 14-Fr silicone tube with a 1-0 silk thread. The silicone tube was then removed, and a colonoscopy was performed to observe the caliber of the stenosis. The success of modeling was judged based on the failure of the colonoscope to pass through the silk suture-ligation site. A colonoscopy confirmed that the rectal stenosis model was successfully established. The abdomen incision was closed using layer-by-layer intermittent suture, 1-0 silk suture for peritoneum and 2-0 silk suture for skin.

### Surgical procedures

Iohexol dilute solution (30% iohexol 50 ml diluted with normal saline 50 ml, 50–80 ml per dog) was injected through the anal cannula to perform rectography to identify the location of rectal stenosis under X-ray. The Y–Z DMR was assembled according to Fig. 1B. Under X-ray monitoring, the “S”-shaped Y–Z DMR gradually crossed the narrow segment of the rectum with close cooperation of the control tube and the two control lines in a serpentine motion by pushing the control tube (Fig. 2A–E). Then, the Y–Z DMR was deformed by pulling the distal control line (Fig. 2F,G), and the “O”-shaped state (shown in Fig. 2H**)** was finally formed.

**Figure 3:**
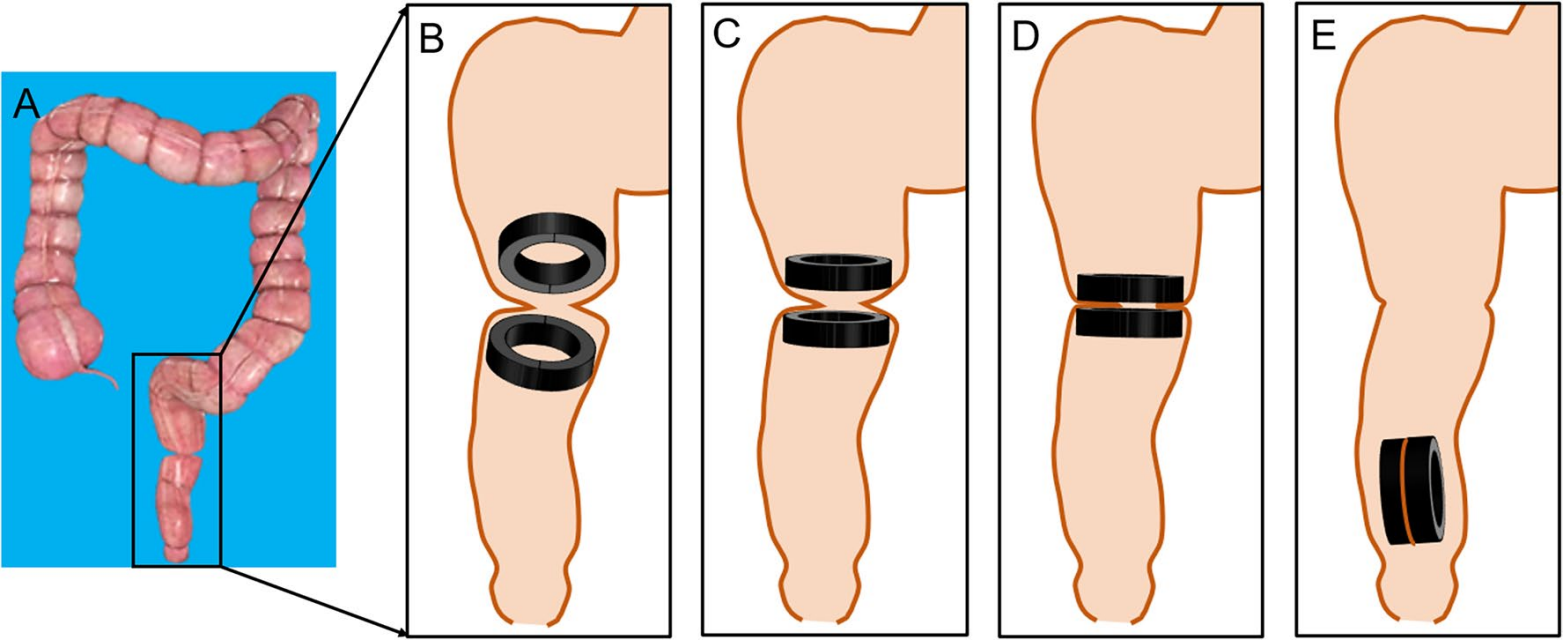
Schematic representation of magnamosis for recanalization of rectal stenosis. (**A**) Schematic representation of rectal stenosis. (**B**) Magnetic rings placed at each end of the rectal stenosis. (**C**) The magnetic rings at both ends of the rectal stenosis attracted to each other. (**D**) The magnetic ring compressed the narrow segment of the rectum. (**E**) The magnetic ring excreted from the narrow segment of the rectum, and rectal anastomosis is established.

The Y–Z DMR assembled into an “O”-shaped state (Fig. 1D) was inserted through the anus to the distal end of the rectal stenosis (Fig. 3A,B). Meanwhile, the magnetic rings located at the proximal and distal ends of the rectal stenosis were attracted together (Fig. 3C). When complete necrosis of the compressed tissue occurred, the magnetic ring detached from the stenosis and passed spontaneously through the anus, after which the patency of the stenosis was restored (Fig. 3D,E).

### Postoperative care

After the magnets at both ends of the rectal stenosis were attracted, a colonoscopy was immediately performed to observe the state of the magnets in the rectum. The dogs were fed in a single cage and administered a non-residue liquid diet after waking from anesthesia. Pethidine was administered intramuscularly every 12 h as an analgesic during the first three postoperative days. The magnet discharge time of each dog was closely observed and accurately recorded. When the Y–Z DMR was excreted, the dogs were fed a normal full-nutrition dog feed. Colonography and colonoscopy were performed immediately after the magnet was discharged to observe the patency of the rectum.

### Burst pressure

Two weeks after removing the magnets, the dogs were euthanized (3% pentobarbital sodium solution 2 ml/kg intravenous rapid infusion), and rectal intestinal specimens were obtained from approximately 10 cm at each end of the anastomosis. The distal rectum was clipped with a vascular clamp, and the proximal rectum was inserted into one end of the catheter and firmly fixed with a silk suture. The other end of the catheter was connected with a modified blood pressure-measuring device. The whole specimen was completely immersed in a 0.9% sodium chloride solution, and the pressure in the intestinal tube was gradually increased. Bursting pressure was recorded when the first bubble at the anastomotic site spilled over the anastomosis.

### Specimen collection and histological analysis

All anastomosis-bearing segments with a sufficient length on either side of the anastomosis were harvested. After gross observation, all samples were immersed in 10% buffered formalin overnight. After fixation, the samples were embedded in paraffin, and 4-μm-thick sections were cut at the anastomosis site. The sections were stained with hematoxylin and eosin (HE) and Masson dye for visualization under light microscopy.

### Statistical analyses

SPSS statistics software v20.0 was used for data analysis. Quantitative data are expressed as the mean ± standard deviation (SD).

## Results

### Rectal stenosis model

**Figure 4:**
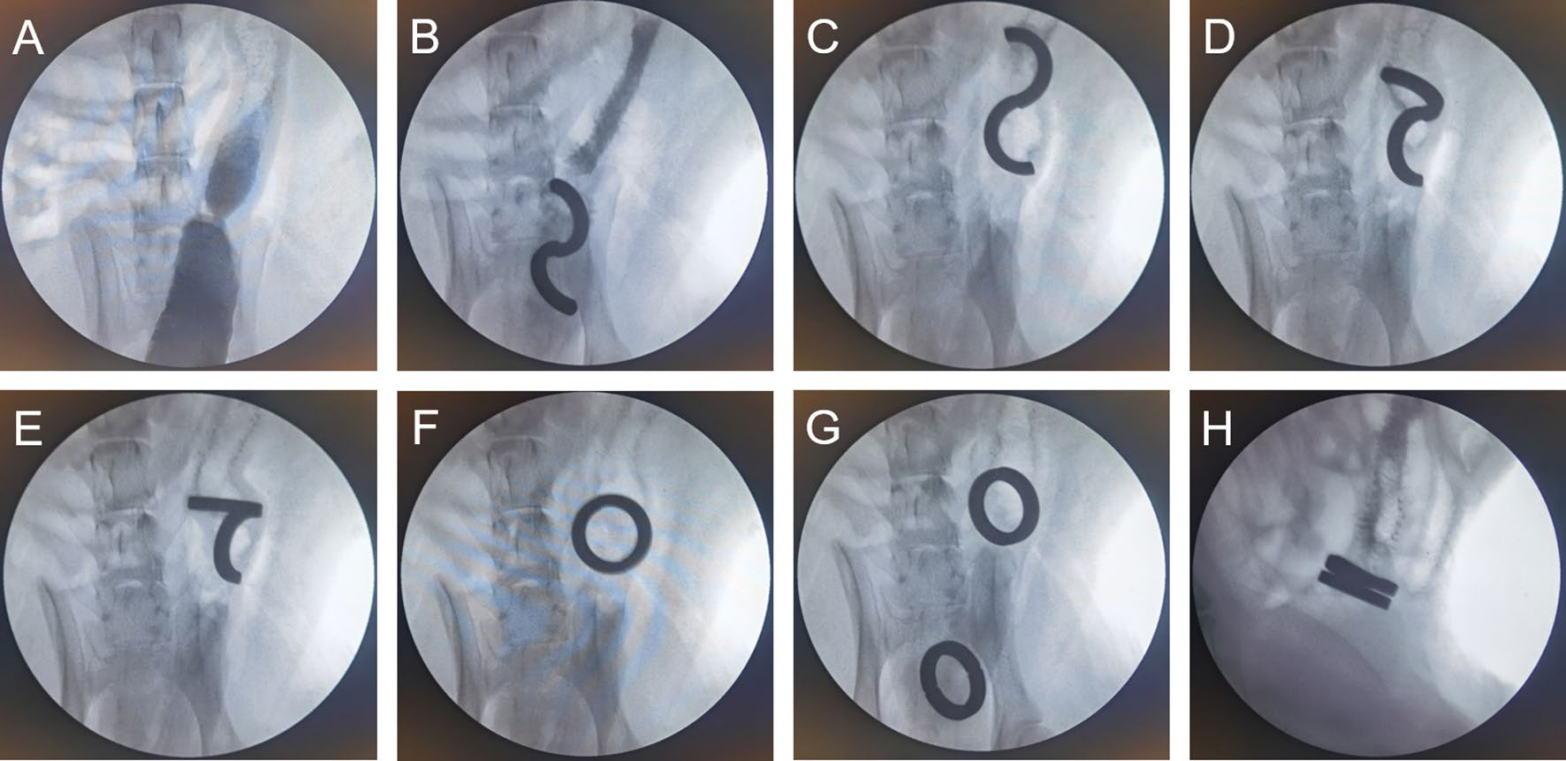
Operational process. (**A**) Rectography demonstrating rectal stenosis. (**B**) The “S”-shaped magnetic ring was inserted into the distal rectum. (**C**) The “S”-shaped magnetic ring was inserted into the proximal rectum. (**D,E**) The “S”-shaped magnetic ring during deformation. (**F**) The magnetic ring in the proximal rectum was completely transformed into an “O”-shape. (**G**) A distal magnetic ring was placed transanally. (**H**) The magnets at both ends of the rectal stenosis attracted to each other.

**Figure 5:**
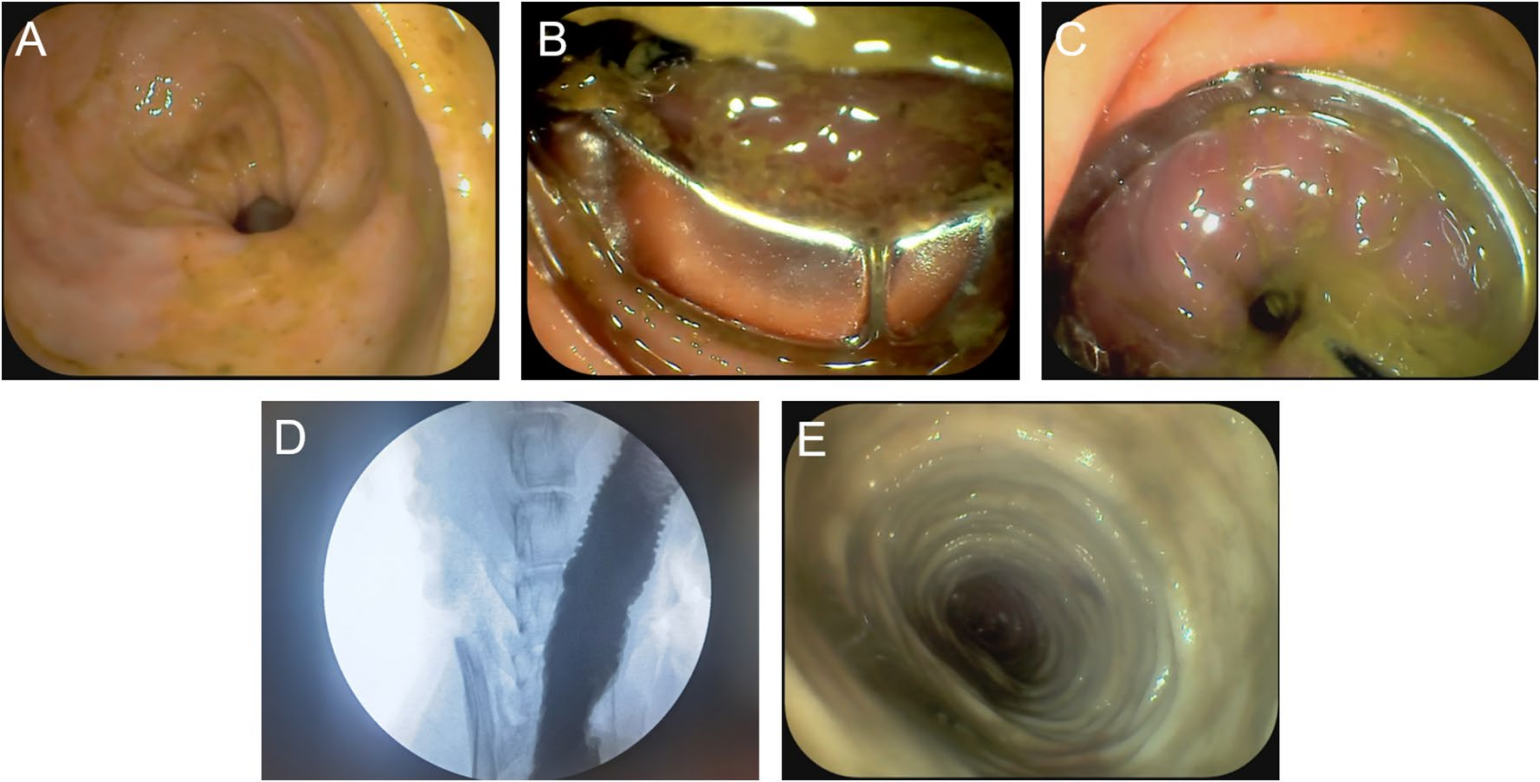
Colonoscopy. (**A**) Rectal stenosis observed by endoscopy. (**B,C**) The magnets at the distal end of the rectum observed endoscopically. (**D**) Rectography was performed after the magnets were excreted. (**E**) Colonoscopy showing good rectal patency after the magnets were excreted.

A rectal stenosis model was successfully established in 8 beagle dogs by partially ligating the rectum with a silk thread under an open abdomen. Colonography revealed that the stenosis was fixed (Fig. 4A), and colonoscopy showed that the inner diameter of the rectum at the stenosis was approximately 4 mm (Fig. 5A). The success rate of the model establishment was 100%, and there were no complications such as bleeding or rectal injury.

### Procedural parameters

The X-ray images showed that the Y–Z DMR in the “S”-shaped state, as guided by the control line and control tube, successfully passed through the constriction portion of the rectum and entered the proximal rectum in a serpentine motion (Fig. 4B,C). The magnetic ring smoothly morphed from an “S” shape to an “O” shape upon adjusting the control tube and control line (Fig. 4D–F). After pushing a magnet through the anus and into the rectal stenosis, an “O”-shaped magnetic ring was positioned at the distal end of the stenosis, and the magnets at the two ends of the stenosis were automatically phase-attracted (Fig. 4G,H). Through colonoscopy, the magnets in the distal rectum were confirmed to be in good working order (Fig. 5B,C). All 8 dogs underwent surgery simultaneously without any complications. There was no magnet displacement, no unintended deformation, and no rectal injury. The average operation time of the magnet installation procedure was 11.75 ± 1.98 min (range 9–15 min).

### Survival rate and postoperative complications

**Figure 6:**
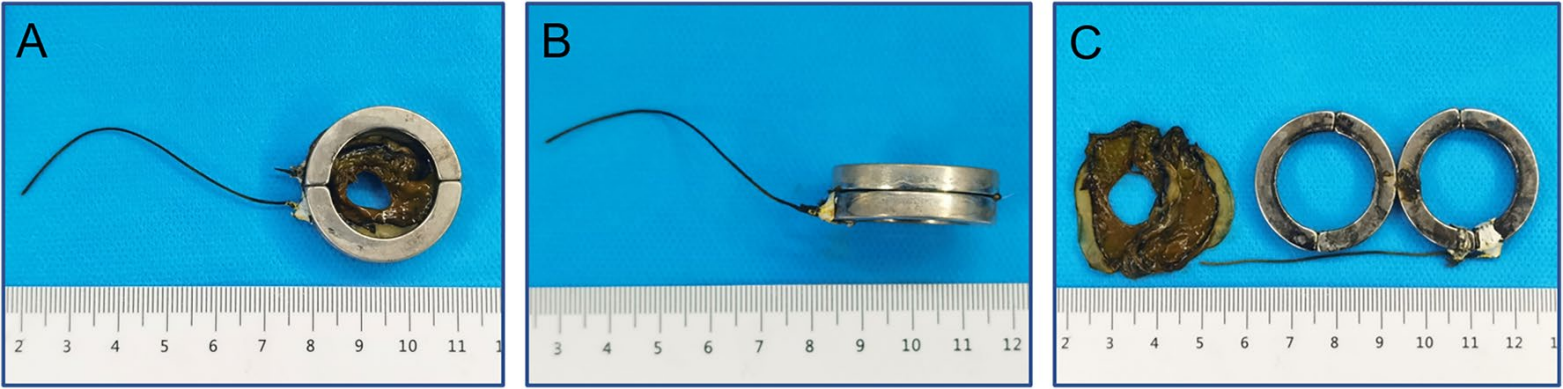
The magnets expelled from the body. (**A,B**) Magnets in the upper and side view. (**C**) Magnets and necrotic rectal tissues.

After the surgery, the health of all 8 dogs was found to be good, and there was no evidence of intestinal obstruction. At 7–10 days post-operation the magnetic rings passed out of the body through the anus. The magnetic rings were in good condition, and the compressed ischemic necrotic intestinal tissues were visible between the rings (Fig. 6A–C). Colonography after magnet removal confirmed that the rectum was completely unblocked (Fig. 5D), while colonoscopy indicated no stenosis in the rectal bowel, no ulcer in the rectal mucosa, and no granulation tissue proliferation (Fig. 5E).

### Bursting pressure

The gross specimens were taken from all experimental animals using a self-modified sphygmomanometer 2 weeks after the magnets were removed. The pressure at the anastomosis site, at which bubbles overflowed, was considered to indicate the bursting pressure of the anastomosis. Since the maximum measurement range for sphygmomanometers is 300 mmHg, the actual pressure can be recorded when the pressure is less than 300 mmHg. However, when the pressure reached the maximum range of the sphygmomanometer, but no bubble overflow was detected at the anastomosis site, the burst pressure was recorded to be > 300 mmHg.

### Gross appearance of anastomosis

**Figure 7:**
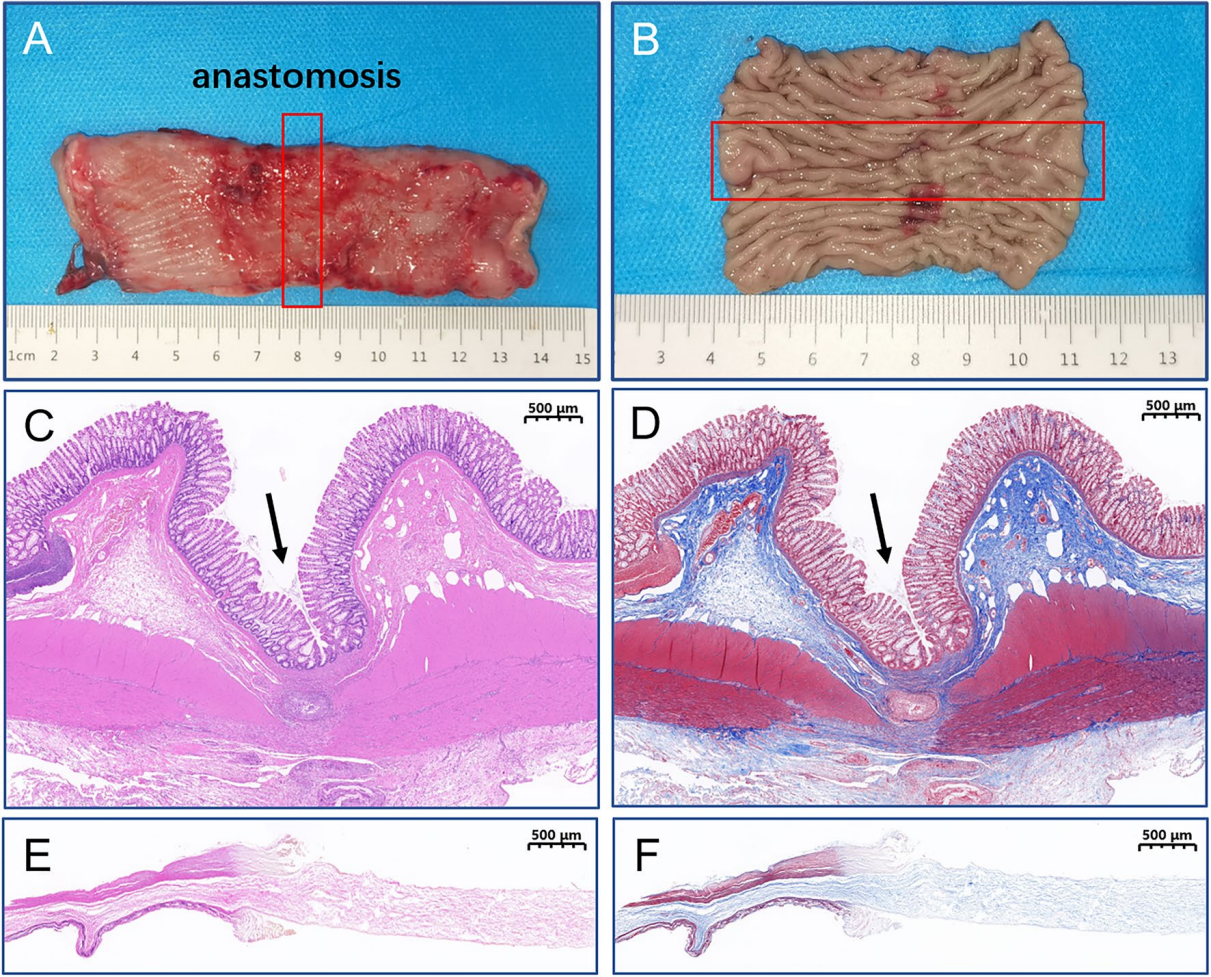
Gross and histological observation of anastomosis. (**A**) The serosal surface of the rectal anastomosis. (**B**) The mucosal surface of the rectal anastomosis. (**C**) The anastomosis stained with HE (2×). (**D**) The anastomosis stained with Masson (2×). (**E,F**) HE and Masson staining of the necrotic rectal tissues.

The serosa layer of the anastomosis showed signs of healing in the gross specimen. The longitudinal resection of the rectum revealed an anastomotic sulcus on the mucosal surface, and the mucosa healed well without hyperplasia or ulceration (Fig. 7A,B). The rectal mucosa and submucosa appeared healthy based on HE and Masson staining of the anastomotic tissues (Fig. 7C,D). The necrotic and exfoliated tissues of the rectal stenosis are depicted in Fig. 7E,F.

## Discussion

More than 40 years have passed since the first magnetic compression anastomosis was performed. Most anastomosis magnets used in the past were cylindrical or ring-shaped. It was discovered, however, that the cylinder or ring structure was inadequate for the anastomosis reconstruction of complex and diverse gastrointestinal stenotic lesions when magnetic anastomosis was combined with endoscopic technology. Significant progress in magnamosis became possible thanks to the idea of a deformable magnetic ring in the design.

When combined with endoscopic technology, a deformable magnetic ring can be passed through the narrow lumen of the digestive tract, and its subsequent deformation can develop a larger compression anastomosis surface and a more optimal anastomotic diameter. These types of clinical requirements informed the development of technologies such as the self-forming magnet (SFM) anastomosis system^22^ and Smart Self-Assembling Magnets for Endoscopy (SAMSEN)^23^. The use of magnetic anastomosis technique to treat rectal stenosis has been documented in various studies^24–26^. The placement of magnetic rings at both ends of the stenosis was easier in these clinical scenarios because the patients had two access points: the trans-stoma approach and the transanal approach. However, there is still a considerable number of patients with rectal AS who do not undergo colectomy or ileostomy. Among the primary challenges presented by the single-access scenario is the impossibility of accommodating the entire magnetic ring within the proximal rectum of the narrow segment. Moreover, it has been reported that AS can recur after magnetic anastomosis recanalization in clinical cases of rectal stenosis^26^. The reason is that, in most cases, as anastomotic stricture is typically caused by hypertrophic scar tissue at the anastomosis. Thus, it is important to “root out” as much of this tissue as possible during the reconstruction of magnetic recanalization anastomosis.

It is challenging to maintain long-term patency when the diameter of the magnetic anastomosis ring is small because there is fewer tissue to remove, and the scar tissue regrows after the surgery. To obtain a long-term stable patency effect, the second-most important procedure involves the use of a magnetic ring with as large a diameter as possible. These two central concerns were considered during the course of the present study. The external control magnetic ring can be placed, and its state can be changed because the lesion site of rectal stenosis is typically close to the location of the anus. Therefore, we advocate for a Y–Z-type magnetic anastomosis ring design. The Y–Z-type magnetic anastomosis ring has the following characteristics: (1) The magnetic anastomosis ring is made up of two semicircle magnetic rings. A small cross-sectional area and the possibility of serpentine movement across the narrow segment is achievable after the two semicircular magnetic rings are attracted to each other, and their axial rotations form an “S” shape. (2) The Y–Z type magnetic anastomosis ring can be deformed from an “S” to an “O” shape when the traction line and control tube are activated, which effectively resolves the issue of passing through the narrow segment. This is because the “S” shape and serpentine motion are optimal for navigating the narrow segment. Therefore, the issue of insertion operation can be ignored when designing the diameter of the magnetic anastomosis ring. Moreover, the diameter of the magnetic anastomosis ring should be close to the inner diameter of the rectum to establish conditions for “eliminating” the anastomotic scar to the greatest extent possible.

We acknowledge that there is a major limitation in our study. The pathological basis of AS after rectal cancer surgery differs significantly from the model of rectal stenosis prepared through partial ligation with a silk thread. Moreover, the feasibility of Y–Z DMR in experimental animals was the primary focus of this study, and the observation time of anastomosis was very short. Long-term anastomosis patency needs to be assessed in future studies to provide a firm basis for clinical transformation and application of Y–Z DMR. In clinical practice, endoscopic balloon dilation, stent implantation, and surgical procedures can be used to treat rectal stenosis through single access. Our recently developed deformable self-assembled magnetic anastomosis ring (DSAMAR) also has the potential to be used in patients with single-pass rectal stenosis^27–29^. This study offers a potential treatment method for such patients.

## Conclusion

In conclusion, the Y–Z type magnetic anastomosis ring can be successfully inserted and properly formed in an animal model. AS following rectal cancer surgery under a single transanal pathway can be effectively treated with magnetic anastomosis technology. This study provides useful exploratory data for further development and application of this technique. With further optimization of the magnetic ring design and the operation path, the Y–Z type magnetic anastomosis ring is expected to be used efficiently in clinical practice.

## Data Availability

The data underlying this article will be shared on reasonable request to the corresponding author. The reporting in the manuscript follows the recommendations in the ARRIVE guidelines.
